# Differential BK channel potentiation by vanzacaftor enantiomers enables therapy for modulator-ineligible people with cystic fibrosis

**DOI:** 10.1172/JCI191824

**Published:** 2025-08-07

**Authors:** Nathalie Baumlin, Sumedha Gunewardena, Scott H. Randell, Frank T. Horrigan, Matthias Salathe

**Affiliations:** 1 Internal Medicine and; 2 Cell Biology and Physiology, University of Kansas Medical Center, Kansas City, Kansas, USA.; 3 Cell Biology and Physiology, University of North Carolina Chapel Hill, Chapel Hill, North Carolina, USA.; 4 Integrative Physiology, Baylor College of Medicine, Houston, Texas, USA.

**Keywords:** Genetics, Pulmonology, Genetic diseases, Potassium channels, Therapeutics

**To the Editor:** Cystic fibrosis transmembrane conductance regulator (CFTR) modulators have significantly improved outcomes for people with cystic fibrosis (pwCF), yet those with unresponsive mutations remain without effective treatments. One promising strategy involves enhancing ion channels alternative to CFTR (reviewed in ref. 1), including apically expressed large conductance, calcium-activated potassium (BK) channels. BK channels, composed of the pore-forming subunit KCNMA1 (Slo1) and the regulatory subunit LRRC26 (γ1), are critical for airway surface hydration and mucociliary transport (MCT). Inhibition or knockdown of these components leads to reduced airway surface liquid, increased mucus concentration, and impaired MCT in CF models in vitro and in vivo (2, 3). BK plays a crucial role in enhancing chloride exit, a phenomenon called apical loop current. Our simulations indicated that small apical increases in potassium conductance (from 0 to 0.002 S/cm^2^) enhance Cl^–^ efflux approximately 3-fold. Reanalyzing airway single cell RNA-seq data from ref. 4, we show KCNMA1, LRRC26, and ANO1 expression in secretory cells (Figure 1A). Among BK β-subunits, only KCNMB2/4 are detected, with KCNMB2 in ciliated cells and KCNMB4 in large airways (Figure 1A), supporting the hypothesis that, in peripheral airways where CF disease starts, apical loop currents between ANO1 and BK, made of KCMNA1/LRRC26, can be enhanced therapeutically.

Our study investigates the restoration of mucociliary function in CF airway epithelia by pharmacologically activating BK channels. Beyond acute BK activation shown by others (5), we found that the CFTR modulator elexacaftor, at concentrations reached in pwCF, potentiates BK activity in normal and CF bronchial epithelial cells approximately 2-fold (Figure 1B). It is unclear if this is clinically meaningful, as pwCF with minimal function variants treated with elexacaftor/tezacaftor/ivacaftor showed, on average, only a marginal, not clinically meaningful FEV1 increase (6) and as expected no sweat chloride level changes.

Others (5) described that vanzacaftor, a new CFTR modulator, acutely activates BK. We therefore explored the effects of vanzacaftor, especially its enantiomers. Both S- and R-vanzacaftor activate BK channels, but with distinct temporal profiles.

S-vanzacaftor increases basal BK activity in *Xenopus* oocytes expressing BK channels (hSlo1±LRRC26) greater than 1,000-fold over control (Figure 1E and Supplemental Figure 2; at –80 mV with 0 Ca^2+^) comparable to saturating Ca^2+^, with detectable activity at concentrations as low as 50 nM (Supplemental Figure 1). Similarly, R-vanzacaftor increases BK activity acutely by 455-fold at 5 μM, much greater than 10 μM elexacaftor (3.170 ± 0.28-fold, Supplemental Figure 1). In CF airway epithelia, S-vanzacaftor acutely activates BK (Figure 1, F and G) and modulates F508del-CFTR (Figure 1C), yet fails to potentiate BK in the long term (Figure 1D, 24 h). In contrast, R-vanzacaftor does not modulate CFTR but activates BK and, more importantly, potentiates BK activity in the long term (Figure 1H), thereby improving MCT in CF cells (Figure 1I and Supplemental Figure 3C). These improvements were related to BK, as KCNMA1 or LRRC26 knockdowns blunt currents and physiological responses (Figure 1, J–L).

The differential effects of enantiomers raise important mechanistic questions. That both activate BK in excised patches with concentration-response curves well fit by Hill coefficients of 2, but not 1 (Supplemental Figure 2E), suggest similar mechanisms of acute action involving direct cooperative channel binding with modest stereoselective efficacy. However, the lack of sustained potentiation by S-vanzacaftor reveals additional differences, potentially involving mechanisms such as desensitization or stereoselective metabolism, which will require additional experiments to resolve. Thus, our data uniquely highlight enantiomer-specific effects of vanzacaftor with long-term therapeutic implications.

In summary, R-vanzacaftor emerges as a promising candidate for pwCF with minimal function mutations, offering a CFTR-independent mechanism to restore mucociliary clearance. Further studies are warranted to elucidate the molecular basis of its sustained BK potentiation and to evaluate clinical efficacy, likely via an inhaled drug delivery route.

## Supplementary Material

Supplemental data

Supporting data values

## Figures and Tables

**Figure 1:**
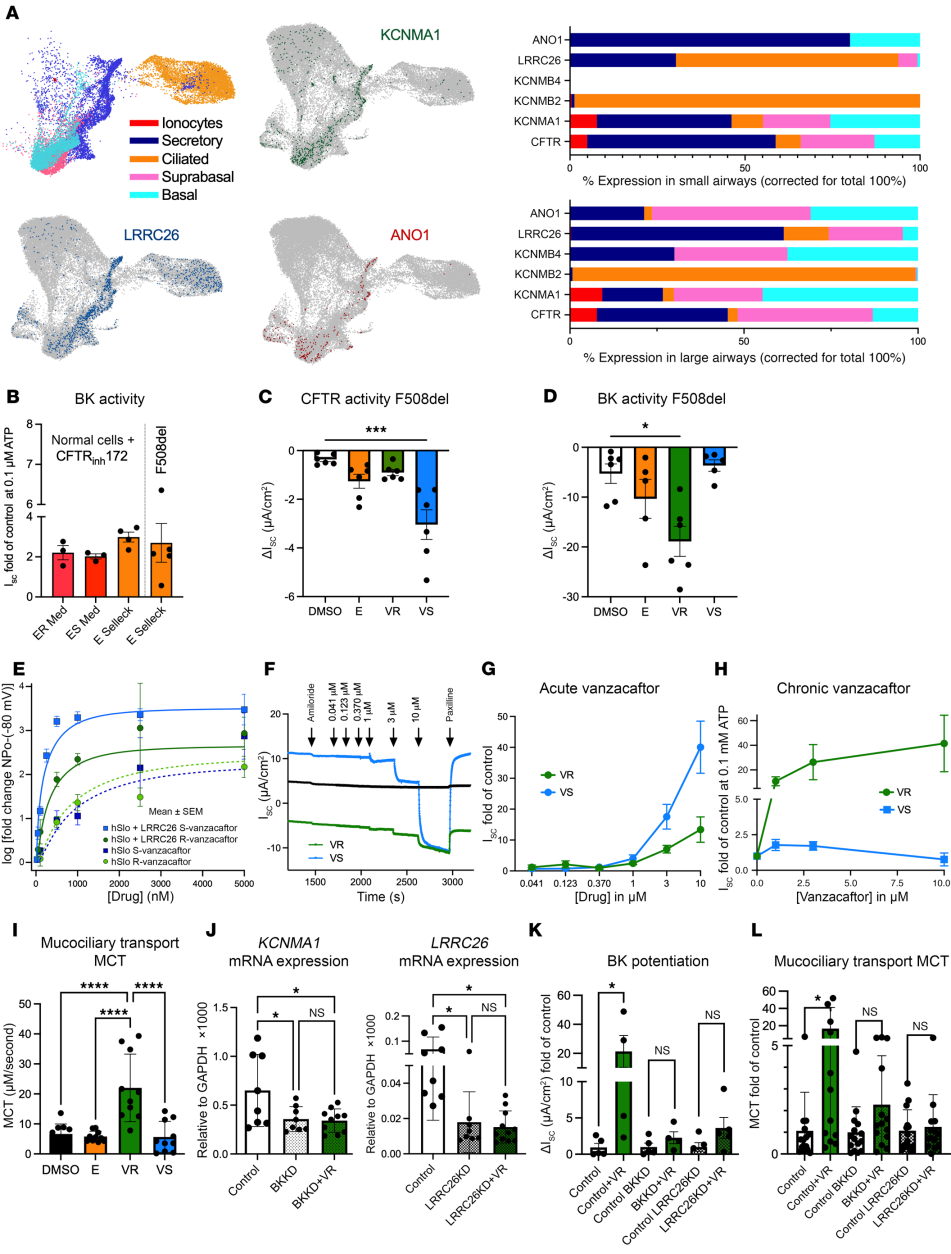
BK channel activation/potentiation by vanzacaftor enantiomers. (**A**) Data reanalyzed from ref. 4. Left: UMAP projections of major cell types. Right: Ion channel expression in large/small airways. (**B**) Fully differentiated, primary normal human bronchial epithelial cells exposed basolaterally to 5 μM elexacaftor (24 hours). To assess BK activity, cells were permeabilized basolaterally in Ussing chambers under a basolateral to apical potassium gradient (3). To increase intracellular calcium, cells were stimulated with 0.1 μM ATP in the acute presence of 10 μM CFTR_inh_172 mimicking minimal function CFTR variants (CFTRMF). Short circuit currents represent basolateral to apical potassium flux. F508delCFBE cells were used without CFTR_inh_172. Differently sourced elexacaftor potentiated BK approximately 2-fold. (**C** and **D**) F508delCFBE cells exposed basolaterally to 5 μM elexacaftor and 5 μM R- or S-vanzacaftor for 24 hours. (**C**) S-vanzacaftor modulates CFTR (***P < 0.05 Friedman; short circuit current inhibition by CFTR_inh_172 after forskolin stimulation). (**D**) R-vanzacaftor potentiates BK (*P < 0.05 Kruskal-Wallis). (**E**) Oocytes; Log or fold-increase in BK (hSlo1±LRRC26) activity (NP_O_ at –80 mV, 0 Ca) over vehicle (mean ± SEM, *n* = 13–25 patches per condition), fit with Hill equations (see Supplemental Figure 2). (**F** and **G**) Acute exposures of CFTRMF cells to vanzacaftor enantiomers after basolateral permeabilization with basolateral to apical potassium gradient (Ussing traces in **F** and summary data in **G**). S-vanzacaftor is the most efficacious BK activator (*n* = 4). (**H**) CFTRMF cells: 24 hour basolateral exposures before testing BK potentiation with ATP (see above) show that R-vanzacaftor is the most efficacious BK potentiator (*n* = 4). (**I**) Only R-vanzacaftor (3 μM basolaterally for 24 hours) improves mucociliary transport (MCT) in CFTRMF cells. ****P < 0.0001 ANOVA and Tukey after passing normality test (*n* = 10). (**J**–**L**) CFTRMF cells with KCNMA1 or LRRC26 knockdown (BKKD, LRRC26KD). (**J**) *KCNMA1* (left) and *LRRC26* (right) mRNA expression (*n* = 8–11). One-way ANOVA / Tukey (left) or Kruskal Wallis (right). (**K**) BK potentiation by 3 μM R-vanzacaftor (24 hour basolaterally) is eliminated by KCNMA1 and LRRC26 KD. (**L**) R-vanzacaftor’s effect on MCT (treatment/DMSO) in CFTRMF (3 μM for 24 hours; *n* = 15). Baselines: 2.9 ± 1.3 μm/s. *P < 0.05 by Mann-Whitney.

## Version 1 08/07/2025

In-Press Preview

## Version 2 10/15/2025

Electronic publication
