# Supplementary material for: Differential BK channel potentiation by vanzacaftor enantiomers enables therapy for modulator-ineligible people with cystic fibrosis
Source: J Clin Invest. 2025 Aug 7;135(20):e191824. doi: 10.1172/JCI191824 (PMC12520667; doi:10.1172/JCI191824)
Supplement: Supplemental data [file jci-135-191824-s119.pdf]

Supplemental Materials for

Differential BK channel potentiation by vanzacaftor enantiomers enables therapy for modulator-ineligible people with cystic fibrosis

Nathalie Baumlin[1], Sumedha Gunewardena[2], Scott H. Randell[3], Frank Horrigan[4], and Matthias Salathe[1]

[1]Department of Internal Medicine and [2]Cell Biology and Physiology, University of Kansas Medical Center.

[3]Department of Cell Biology and Physiology, University of North Carolina Chapel Hill.

[4]Department of Integrative Physiology, Baylor College of Medicine.

Correspondence: Matthias Salathe, Department of Internal Medicine, University of Kansas Medical Center, 3901 Rainbow Blvd., 2025 Murphy, Kansas City, KS 66160, USA. Phone: +1 913 588 6000, msalathe@kumc.edu.

Supplemental methods

Sex as a biological variant

Given the small number of available donors, female and male differences (sex as a variable) was not assessed. However, supplemental table S1 provides an overview of all donors.

Lungs

Human bronchial epithelial cells were harvested from lungs of never-smoking donors (NHBE) with no known lung diseases that were deemed not suited for transplant. These lungs were recovered for research by organ procurement agencies, including the Life Alliance Organ Recovery Agency at the University of Miami (Miami, FL), LifeCenter Northwest (Seattle, WA), the Midwest Transplant Network (Kansas City, KS) and Nevada Donor Network (Las Vegas, NV). Consents for organ donation were obtained by the organ procurement agencies. Since these materials were procured from deceased individuals, the University of Kansas Institutional Review Board confirmed that cell and tissue use are not considered human subject research. NHBE cell information is presented in supplemental table S1. Human cystic fibrosis bronchial epithelial cells with at least one copy of F508del (CFBEF508del) were harvested from appropriately consented patients undergoing lung transplantation, approved by the University of Miami's IRB, with an MTA to the University of Kansas Medical Center. Lungs from one deceased CF donor (CFBE-R1162X) were procured through LifeCenter Northwest. Furthermore, the CF Foundation's biorepository provided hBE cells with G542X/R553X and K710X/L467P variants (passage 1 and 2). Lung donor information is presented in supplemental table S1.

Cell culture

NHBE and CFBE cells were cultured as described (1). Briefly, cells were thawed and plated into a 10 cm dish in PneumaCult Ex media for 4-5 days. Then cells were further expanded in BEGM media (2), before seeding on 1.13 cm^2 Transwells in air-liquid interface (ALI) media (2) and kept submerged for 4-5 days before exposing them to air. The cells were cultured in ALI

media for another 4-6 weeks, replacing media only in the basolateral chamber and adding PBS in apical chamber to wash and remove mucus accumulations; this process was done every other day. After 4-6 weeks, the cells displayed cilia beating and transport of particles and debris.

Cell culture of BMI airway cells (3): semi-immortalized CFBE cells

CF hTERT/ BMI-1 cells (3) carrying minimal function CFTR variants (BMI-G542X and BMI-W1282X) were first expanded in Pneumacult Ex basal media in a 10 cm dish and passaged onto 1.13 cm^2 Transwells and cultured with the same methods as primary NHBE and CFBE cells (see above).

Single Cell RNA Seq Data Re-Analysis from (4)

Data Normalization and Integration: Data normalization was performed using the SCTransform (5) function in the Seurat R package v4 (6) with default parameters. To integrate data across samples, Harmony software (7) was employed, allowing for batch effect correction and unified downstream analysis. *Dimensionality Reduction, Clustering, and Visualization:* Dimensionality reduction, clustering, and visualization were performed using Seurat (6). Clustering stability and resolution selection were guided by the Clustree package (8), with the top-level partitioning set at a resolution of 0.4, yielding 16 clusters. *Cell Type Annotation:* Cell type identification for each cluster was performed using the SingleR package (9). Annotations were based on the Human Primary Cell Atlas (10) (HPCA) reference dataset and pre-labeled single-cell datasets from studies GSE134174 (11), GSE160664 (4), and GSE160673 (4). *Gene Expression Analysis:* Conserved genes across clusters and treatment groups were identified using the FindConservedMarkers function in Seurat. Differentially expressed genes between treatment groups and clusters were identified using the FindMarkers function in Seurat.

***Xenopus* oocytes**

BK channels consisting of the human pore forming subunit (hSlo1) alone or with LRRC26 were expressed in *Xenopus* oocytes. Oocytes were injected with ~3 ng of cRNA incubated at 19°

C and studied 2–7 days after injection. To assure saturation of channels with LRRC26 subunits a 7:1 molar ratio of LRRC26:hSlo1 cRNA was injected. Use of *Xenopus Laevis* was approved by the Institutional Animal Care and Use Committee of Baylor College of Medicine.

KCNMA1 and LRRC26 knockdown using lentiviruses

Lentiviruses were produced as previously described (12) using KCNMA1 knockdown and LRRC26 knockdown plasmid DNA (13, 14). Cells were seeded on Transwells at $2x10^5$ cells and lentiviruses were added (50 ng per $2x10^5$ cells) in 500 µL BEGM media (2), containing 2 µg/mL polybrene (MilliporeSigma cat# H9268) and incubated overnight. After incubation, the apical media was removed and replaced with ALI media (2); 1 mL of ALI media was also replaced basolaterally. Twenty-four hours later, the media was replaced again, but this time with addition of puromycin at 1 µg/mL. The subsequent steps of culturing were the same as for non-infected NHBE or CFBE cells (see above) with the exception that puromycin remained in ALI media until the experiments were carried out.

Treatments

In Fig. 1B, NHBE and CFBE-F508del were treated with elexacaftor (E) from different suppliers at 5 µM for 24h. $CFTR_{inh}172$ was added only into the Ussing chambers at recording where indicated. ER Med: R-elexacaftor from MedChemExpress cat# HY-111772A; ES Med: S-elexacaftor from MedChemExpress cat# HY-111772; E Selleck: elexacaftor from Selleckchem cat# S8851. In Fig. D-M: E – elexacaftor from Selleckchem, VR - R-vanzacaftor from MedChemExpress cat# HY-145603A and VS – S-vanzacaftor from MedChemExpress cat# HY-145603. In Fig. 1D: elexacaftor and vanzacaftor at 5 µM for 24h. In Fig. 1I/L/M, elexacaftor and vanzacaftor enantiomers at 3 µM for 24h. These concentrations of elexacaftor and S-vanzacaftor are reached at steady state in pwCF using recommended daily doses, i.e., 200 mg elexacaftor and 20 mg S-vanzacaftor (15-17).

Ussing chamber for CFTR and BK activities

In all experiments, cystic fibrosis transmembrane conductance regulator (CFTR) and large conductance, Ca^{2+}-activated, and voltage-dependent K^+ channel (BK) activities were measured in Ussing chambers using the same protocol and buffers published before (1). While CFTR follows typical Ussing chamber protocols with consecutive exposures to amiloride (10 µM, MilliporeSigma cat# A7410), forskolin (10 µM, MilliporeSigma cat# F3917), $CFTR_{inh}$172 (10 µM, MilliporeSigma cat# C2992), under a basolateral to apical chloride gradient. CFTR activity is represented by the delta-short-circuit current of $CFTR_{inh}$172 inhibition. BK-related potassium currents were assessed as follows: cells were mounted in Ussing chambers with basolateral membranes permeabilized (10 µM nigericin Tocris cat# 4312, 10 µM valinomycin Tocris cat# 3373 and 20 µM amphotericin B MilliporeSigma cat# 2411) under a basolateral to apical potassium gradient. The cells were then stimulated with ATP to increase intracellular calcium, and the resulting short circuit currents were measured, representing potassium flux as published by us before (13, 14, 18-21). In all Figures measuring BK potentiation by 24h exposures to basolateral elexacaftor and/or vanzacaftor, BK-related currents were recorded using increasing concentrations of ATP: 0.01 µM, 0.1 µM, 1 µM, and 10 µM. To simplify, most figures will depict BK activities in the graphs at stimulations with 0.1 µM ATP. Instead of using ATP as stimulation, BK activities were also measure in response to acute vanzacaftor exposures (increasing concentrations: 0.041 µM, 0.123 µM, 0.370 µM, 1 µM, 3 µM, and 10 µM).To show that the recorded currents were solely from BK channels, we used paxilline, a selective BK blocker (22) at 10 µM (MilliporeSigma cat# P2928) and BK KCNMA1 and LRRC26 knockdowns.

Mucociliary transport measurements (MCT)

Carboxylate-Modified Microspheres purchased as FluoSpheres™ (cat# F8823) were from ThermoFisher Scientific and diluted 1/10,000 in PBS. Twenty-four hours after modulator treatment, MCT rates were recorded by apically applying 10 µL of the diluted microspheres and incubating for 10-30 min at 37 °C in 5% CO_2, allowing the beads to equilibrate and get immobilized

in the controls. After incubation, the velocity for each treatment was recorded every 2 s for 20 s at an emission of 515 nm at three locations on the Transwell using an inverted microscope (Zeiss Axiovert 200M) with a 20x long distance objective and the software MetaMorph. The Manual Tracking ImageJ plugin was used to analyze and quantify the results.

Patch Clamp Electrophysiology

Patch Clamp Electrophysiology was performed as previously described (23). Briefly, patches were excised from *Xenopus* oocytes expressing BK channels in the inside-out configuration at room temperature (21–24°C) and currents recorded with an Axopatch 200B amplifier (Axon Instruments). Currents were filtered at 20 kHz with an 8-pole Bessel filter (Frequency Device, Inc.) and sampled at 50 kHz with an ITC-18 A/D converter (Instrutech), using PATCHMASTER acquisition software (HEKA), and Igor Pro software (WaveMetrics, Inc., Lake Oswego, OR) for graphing and data analysis. Patch pipettes with a resistance of 2-4 MΩ were pulled from PG10150-4 glass (World Precision Instruments) and coated with wax (KERR Sticky Wax). The external pipette solution contained (in mM) 140 K-methanesulfonic acid (K-MES), 2 $MgCl_2$, 6 HCl, 20 HEPES. The internal '0 Ca^{2+}' bath solution contained 140 K-MES, 10 HCL, 20 HEPES, and 5 EGTA, reducing free Ca^{2+} to an estimated 0.8 nM. The pH of all solutions was adjusted to 7.2 with MES. Currents were recorded at -80 mV in the absence of Ca^{2+} to approximate resting conditions in non-excitable cells. Activity (NP_o) was measured in patches containing tens to hundreds of channels over 10 s intervals during steady-state recordings of 30-180 s duration and determined from all-point amplitude histograms by measuring the fraction of time spent (P_K) at each open level (K) using a half-amplitude criterion and summing their contributions ($NPo = \sum KP_K$). Drugs were applied at increasing concentrations by exchanging the bath solution (~10 volumes in 1 min, n = 1 to 3 drug concentrations per patch)). Fold-changes in activity were determined by comparing NPo (mean±SEM, from n = 3-18, 10 s intervals) in the presence of drug to that for the vehicle

control. NPo at higher drug concentrations (≥ 500 nM) were typically measured following a 30-180 s delay to allow activity to reach a steady-state.

Quantitative PCR

CFBE cells were lysed and total RNA isolated using the E.Z.N.A.® Total RNA Kit (Omega Bio-tek). qPCR was performed using TaqMan Gene Expression Assays (ThermoFisher Scientific) for *KCNMA1* (cat# Hs00266938_m1) and *LRRC26* (cat# Hs02385555_g1) and normalized to reference gene *GAPDH (cat#* 4352934E) as described (1) using a CFX96™ real-time PCR detection system (Bio-Rad Laboratories).

Statistical analysis and Data Availability

Specific statistical tests were used depending on whether or not the data passed Shapiro-Wilk normality testing. These tests are included in the figure legends.

Data are available in a supplemental excel sheet. Additional data are available from the authors upon request.

Acknowledgement

This work was supported by the Cystic Fibrosis Foundation (SALATHG240 to MS and RANDEL20XX2 to SHR). We thank John S. Dennis for technical assistance.

Supplemental Table S1

	Sex	Age	CFTR mutations
CF385	Male	38	F508del/F508del
CF663	Female	17	F508del/F508del
CF653	Female	36	F508del/F508del
KC49	Male	30	F508del/F508del
CF582	Male	54	F508del/F508del
CFL10	Male	23	F508del/F508del
CF693	Female	17	F508del/R1162X
CF228	Female	25	F508del/G542X
CFLCNW25	Female	21	R1162X/unknown minimal function
CFF Repository			K710X/L467P
CFF Repository			G542X/R553X
KC38	Female	50	WT nonsmoker
LCNW99	Male	44	WT nonsmoker
LCNW172	Male	60	WT nonsmoker
BMI-G542X UNCCF13T	Female	19	G542X/G542X
BMI-W1282X UNCCF9T	Female	42	W1282X/W1282X

Supplemental Figures

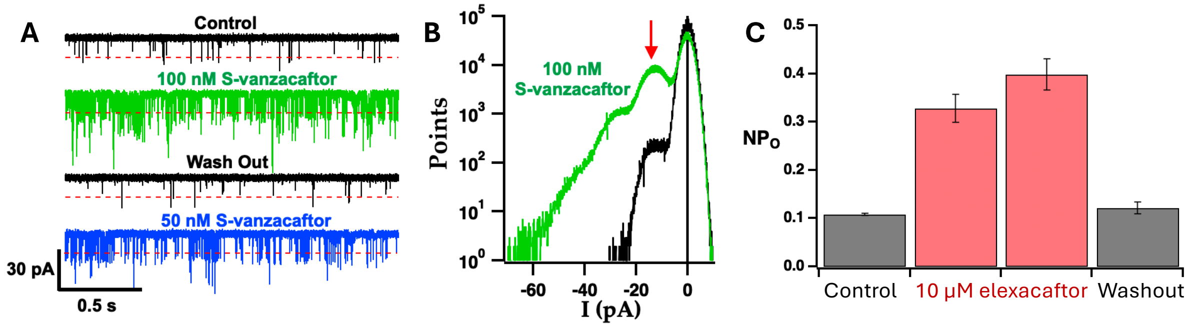

Figure S1. Acute activation of heterologously expressed BK channels by S-vanzacaftor and elexacaftor. (**A**) Steady state BK channel current in response to alternative solution exchange with S-vanzacaftor or vehicle control (0.1% DMSO) showing reversible activation at nanomolar concentrations. Currents were measured by patch clamp electrophysiology (-80 mV, 0 Ca^{2+}) from excised inside-out patches of *Xenopus* oocytes expressing human BK Slo1 and LRRC26 subunits. (**B**) All point amplitude histograms from (**A**) for control and 100 nM S-vanzacaftor (30 s recordings) show an increase in activity without change in single channel current amplitude (arrow, and dashed lines in **A**), consistent with BK channel activation. (**C**) Increased hSlo1/LRRC26 BK channel activity (NPo) by elexacaftor determined from all point histograms (mean±SEM, n=3 from 10 s recordings per condition) during sequential perfusion and washout of 10 μM elexacaftor.

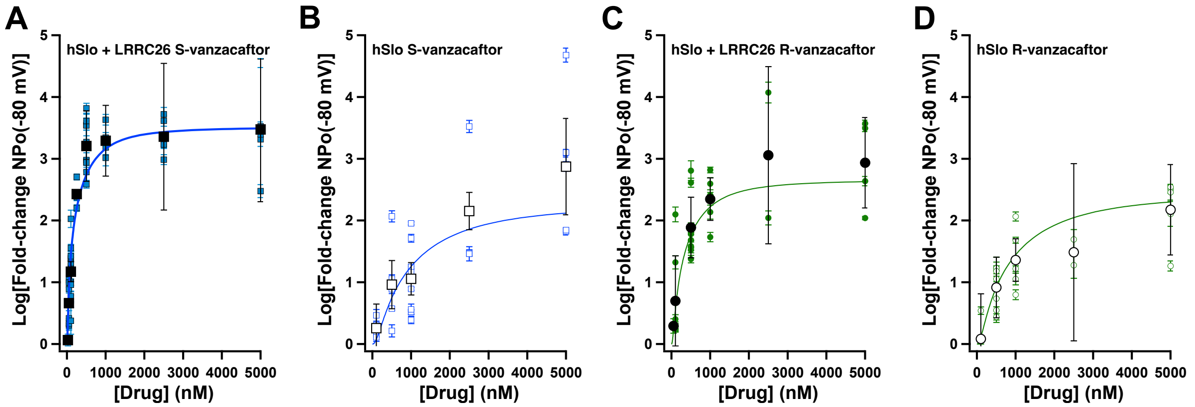

E

			EC50 (nM)		Amplitude (fold-change)	
		N	Mean	SD	Mean	SD
hSlo+LRRC26	S-vanzacaftor	2	1114	44	3256	217
hSlo+LRRC26	R-vanzacaftor	2	1321	49	455	23
hSlo	S-vanzacaftor	2	3102	123	181	12
hSlo	R-vanzacaftor	2	3004	185	270	27

F

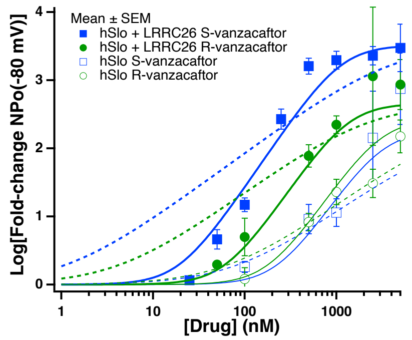

Figure S2. Acute activation of heterologously expressed BK channels by S-and R-vanzacaftor. (**A,B**) BK channel activity (log fold increase in NPo vs control, 0.1% DMSO) in response to S-vanzacaftor or (**C,D**) or R-vanzacaftor. Currents were measured by patch clamp electrophysiology (-80 mV, 0 Ca^{2+}) in excised inside-out patches from *Xenopus* oocytes expressing human BK Slo1 ± LRRC26 subunits. Measurements from individual patches are plotted (mean ± SEM) together with the mean ± SD of all measurements at the same concentration (black symbols). Concentration-response relations were fit (curves) to individual data points using Hill equations with a Hill coefficient of N=2 and other parameters allowed to vary (**E**). The number of patches and oocytes used for each data set were A(25,19); B(11,6); C(17,11); D(13,8). (**F**) Concentration-response relations plotted on a log-log scale are well fit with N=2 (solid curves), but not by a non-cooperative mechanism (N=1, dashed curves) which fails to account for the steepness of the relations.

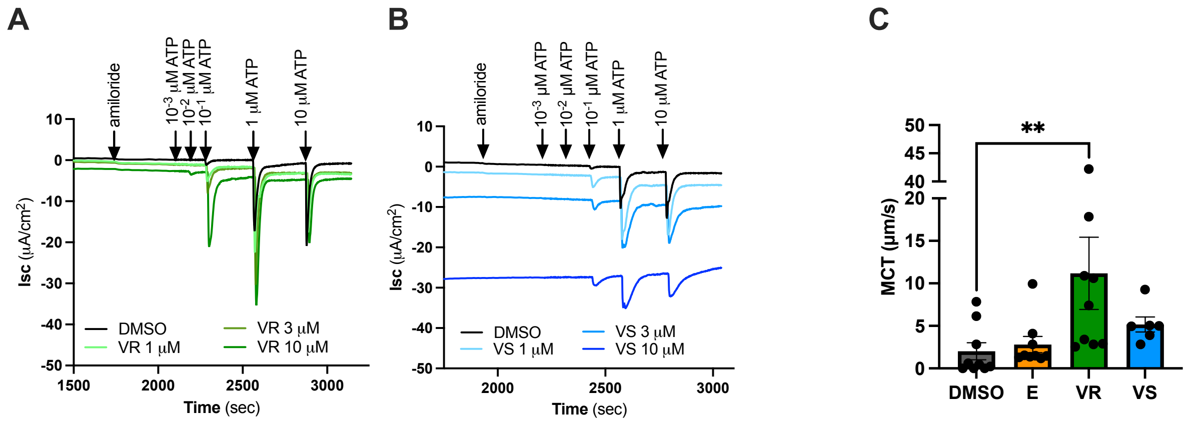

Figure S3. BK potentiation: Ussing traces and quantification using BMI-G542X cells as well as mucociliary transport (MCT) in homozygous F508del cells. (**A-C**) Representative Ussing chamber traces of BK currents in cells exposed for 24h basolaterally to (**A**) R-vanzacaftor or (**B**) S-vanzacaftor. (**C**) Mucociliary transport in homozygous F508del CFBE cells ($n \geq 6$ **$p<0.05$ by Kruskal-Wallis.

References

1. Kim MD, Bengtson CD, Yoshida M, Niloy AJ, Dennis JS, Baumlin N, et al. Losartan ameliorates TGF-beta1-induced CFTR dysfunction and improves correction by cystic fibrosis modulator therapies. *J Clin Invest.* 2022;132(11).
2. Fulcher ML, and Randell SH. Human nasal and tracheo-bronchial respiratory epithelial cell culture. *Methods Mol Biol.* 2013;945:109-21.
3. Lee RE, Lewis CA, He L, Bulik-Sullivan EC, Gallant SC, Mascenik TM, et al. Small-molecule eRF3a degraders rescue CFTR nonsense mutations by promoting premature termination codon readthrough. *J Clin Invest.* 2022;132(18).
4. Okuda K, Dang H, Kobayashi Y, Carraro G, Nakano S, Chen G, et al. Secretory Cells Dominate Airway CFTR Expression and Function in Human Airway Superficial Epithelia. *Am J Respir Crit Care Med.* 2021;203(10):1275-89.
5. Stuart T, Butler A, Hoffman P, Hafemeister C, Papalexi E, Mauck WM, 3rd, et al. Comprehensive Integration of Single-Cell Data. *Cell.* 2019;177(7):1888-902 e21.
6. Hao Y, Hao S, Andersen-Nissen E, Mauck WM, 3rd, Zheng S, Butler A, et al. Integrated analysis of multimodal single-cell data. *Cell.* 2021;184(13):3573-87 e29.
7. Korsunsky I, Millard N, Fan J, Slowikowski K, Zhang F, Wei K, et al. Fast, sensitive and accurate integration of single-cell data with Harmony. *Nature methods.* 2019;16(12):1289-96.
8. Zappia L, and Oshlack A. Clustering trees: a visualization for evaluating clusterings at multiple resolutions. *Gigascience.* 2018;7(7).
9. Aran D, Looney AP, Liu L, Wu E, Fong V, Hsu A, et al. Reference-based analysis of lung single-cell sequencing reveals a transitional profibrotic macrophage. *Nat Immunol.* 2019;20(2):163-72.
10. Regev A, Teichmann SA, Lander ES, Amit I, Benoist C, Birney E, et al. The Human Cell Atlas. *Elife.* 2017;6.
11. Goldfarbmuren KC, Jackson ND, Sajuthi SP, Dyjack N, Li KS, Rios CL, et al. Dissecting the cellular specificity of smoking effects and reconstructing lineages in the human airway epithelium. *Nat Commun.* 2020;11(1):2485.
12. Baumlin-Schmid N, Salathe M, and Fregien NL. Optimal Lentivirus Production and Cell Culture Conditions Necessary to Successfully Transduce Primary Human Bronchial Epithelial Cells. *J Vis Exp.* 2016(113).
13. Manzanares D, Gonzalez C, Ivonnet P, Chen RS, Valencia-Gattas M, Conner GE, et al. Functional apical large conductance, Ca2+-activated, and voltage-dependent K+ channels are required for maintenance of airway surface liquid volume. *J Biol Chem.* 2011;286(22):19830-9.
14. Manzanares D, Krick S, Baumlin N, Dennis JS, Tyrrell J, Tarran R, et al. Airway Surface Dehydration by Transforming Growth Factor beta (TGF-beta) in Cystic Fibrosis Is Due to Decreased Function of a Voltage-dependent Potassium Channel and Can Be Rescued by the Drug Pirfenidone. *J Biol Chem.* 2015;290(42):25710-6.
15. Vertex. A Study of VX-445 in Healthy Subjects and Subjects With Cystic Fibrosis. https://classic.clinicaltrials.gov/show/NCT03227471. Accessed 12/04/2023.
16. Keating D, Marigowda G, Burr L, Daines C, Mall MA, McKone EF, et al. VX-445-Tezacaftor-Ivacaftor in Patients with Cystic Fibrosis and One or Two Phe508del Alleles. *N Engl J Med.* 2018;379(17):1612-20.
17. Vertex. A Study to Evaluate the Safety and Efficacy of VX-121 Combination Therapy in Subjects With Cystic Fibrosis. https://clinicaltrials.gov/study/NCT03912233?tab=results. Accessed 10/3/2024, 2024.

18. Bengtson CD, Kim MD, Anabtawi A, He J, Dennis JS, Miller S, et al. Hyperglycaemia in cystic fibrosis adversely affects BK channel function critical for mucus clearance. *Eur Respir J.* 2021;57(1).
19. Kim MD, Chung S, Baumlin N, Sun L, Silswal N, Dennis JS, et al. E-cigarette aerosols of propylene glycol impair BK channel activity and parameters of mucociliary function. *Am J Physiol Lung Cell Mol Physiol.* 2023;324(4):L468-L79.
20. Manzanares D, Srinivasan M, Salathe ST, Ivonnet P, Baumlin N, Dennis JS, et al. IFN-gamma-mediated reduction of large-conductance, Ca2+-activated, voltage-dependent K+ (BK) channel activity in airway epithelial cells leads to mucociliary dysfunction. *Am J Physiol Lung Cell Mol Physiol.* 2014;306(5):L453-62.
21. Sailland J, Grosche A, Baumlin N, Dennis JS, Schmid A, Krick S, et al. Role of Smad3 and p38 Signalling in Cigarette Smoke-induced CFTR and BK dysfunction in Primary Human Bronchial Airway Epithelial Cells. *Sci Rep.* 2017;7(1):10506.
22. Zhou Y, and Lingle CJ. Paxilline inhibits BK channels by an almost exclusively closed-channel block mechanism. *J Gen Physiol.* 2014;144(5):415-40.
23. Sun L, and Horrigan FT. A gating lever and molecular logic gate that couple voltage and calcium sensor activation to opening in BK potassium channels. *Sci Adv.* 2022;8(50):eabq5772.
